# Editorial: Biomarkers from multi-tracer and multi-modal neuroimaging in age-related neurodegenerative diseases

**DOI:** 10.3389/fnagi.2022.961718

**Published:** 2022-08-03

**Authors:** Binbin Nie

**Affiliations:** ^1^Beijing Engineering Research Center of Radiographic Techniques and Equipment, Institute of High Energy Physics, Chinese Academy of Sciences, Beijing, China; ^2^School of Nuclear Science and Technology, University of Chinese Academy of Sciences, Beijing, China

**Keywords:** deep learning, neurodegenerative diseases, heterogeneous, magnetic resonance imaging, positron emission tomography

Editorial on the Research Topic

Biomarkers from multi-tracer and multi-modal neuroimaging in age-related neurodegenerative diseases

With the progress of neuroimaging methods, more neurodegenerative diseases have been revealed to have heterogeneous phenotypes and stages (Leyton et al., 2011; Thenganatt and Jankovic, 2014; Graff-Radford et al., 2021). This underlying heterogeneity influences the precision of diagnosis and subsequent medical treatment. In this Research Topic on “*Biomarkers from Multi-tracer and Multi-modal Neuroimaging in Age-related Neurodegenerative Diseases*,” researchers have contributed unique opinions and solutions for this issue. For example, using blood oxygen level-dependent (BOLD) functional magnetic resonance imaging (fMRI), Sheng et al. studied the heterogeneous stages of Parkinson's disease by exploring the altered cortical cholinergic network, while Li et al. investigated the alterations of regional homogeneity. Chiu et al. designed a composite scale to improve the diagnostic accuracy of heterogeneous dementia, differentiating Lewy body dementia (DLB) from Alzheimer's Disease (AD).

Apart from traditional statistical methods, as a state-of-the-art method, deep learning (DL) methods have the leading advantage of exploiting hierarchical feature representations, instead of human-designed features by the expert's understanding of the domain (LeCun et al., 2015; Litjens et al., 2017). The DL might therefore be a better method to discover the more heterogeneous patterns of different neurodegenerative diseases.

DL methods can help to improve diagnosis and predictive accuracy. Qu et al. reviewed the performance of the generative adversarial network (GAN) in the diagnosis of AD. Zhou et al. evaluated the deep-learning radiomics (DLR) method for predicting the conversion of mild cognitive impairment (MCI) to AD. The performance of pattern recognition of these models could both exploit the mutual information among different modalities and detect the heterogeneous disease patterns in neuroimaging.

DL methods can also be inspired by other algorithms for their unique characteristics. Some unsupervised methods in machine learning have also shown promising effects. (Díaz-Álvarez et al., 2022) used a machine learning algorithm with genetic algorithms, K-nearest neighbor, and BayesNet Naives to distinguish AD and frontotemporal dementia (FTD). The application of graph theory can lead to the combination of graph neural networks. Wei et al. and Zhang T. et al. have addressed graph characteristics among the regional neuroimaging biomarkers of MCI and AD.

Apart from MCI and AD dementia, Zhang et al. (2021) focused on uncertain cases of memory impairment. The use of the DL method based on ^18^F-fluorodeoxyglucose (FDG) positron emission tomography (PET) can help to distinguish real AD-related pathology from fake memory impairment caused by a depressed mental state. This classification between heterogeneous causes could lead to less misdiagnosis and inappropriate treatment.

Although studies have made much progress in the application of DL among heterogeneous neurodegenerative diseases, some questions are still waiting to be addressed in the future. First, reliable statistical results should also be presented along with the DL results of the disease heterogeneity. Second, after detecting the heterogeneity by innovative methods, more non-imaging information like neuropsychological tests, genetics, and demography can be combined to detect more related features. Finally, the explanation of the DL models should be addressed further through the purposive design of model structures or experiments.

## Author contributions

BN were responsible for the study concept and wrote the manuscript.

## Funding

This work was financially supported by the National Natural Science Foundation of China (12175268 and 11975249).

## Conflict of interest

The author declares that the research was conducted in the absence of any commercial or financial relationships that could be construed as a potential conflict of interest.

## Publisher's note

All claims expressed in this article are solely those of the authors and do not necessarily represent those of their affiliated organizations, or those of the publisher, the editors and the reviewers. Any product that may be evaluated in this article, or claim that may be made by its manufacturer, is not guaranteed or endorsed by the publisher.
